# Presymptomatic Detection and Monitoring of Alzheimer’s Disease Using a Multi‐Disease Diagnostic Platform Employing Autoantibodies as Blood‐based Biomarkers

**DOI:** 10.1002/alz.087141

**Published:** 2025-01-09

**Authors:** Cassandra DeMarshall, Jeffrey Viviano, Mert Sahin, Robert Nagele, the Parkinson’s Study Group (PSG), and the Alzheimer’s Disease Neuroimaging Initiative (ADNI)

**Affiliations:** ^1^ Durin Technologies Inc., Mullica Hill, NJ USA; ^2^ New Jersey Institute for Successful Aging, Rowan University, Stratford, NJ USA

## Abstract

**Background:**

It is well known that Alzheimer’s disease (AD)‐related pathological changes can begin up to a decade before patients experience telltale symptoms, yet presymptomatic detection remains an unmet goal. In the present study, we demonstrate the utility of a panel of autoantibodies capable of detecting AD‐related pathological processes at the earliest points along the AD continuum, including presymptomatic AD (up to 10 years before onset of symptoms) and prodromal AD (mild cognitive impairment, MCI).

**Method:**

Utilizing Duritect‐AD^TM^, a blood‐based test employing a customized panel of autoantibody biomarkers and Luminex xMAP® technology, sera from ADNI subjects with confirmed presymptomatic or prodromal AD (MCI) were screened to detect the presence of AD‐related pathology. Autoantibodies in this panel showing consistently increased titers in MCI and presymptomatic AD relative to controls were evaluated using Random Forest and Receiver Operating Characteristic curves for their ability to distinguish diseased subjects from age‐ and sex‐matched controls, as well as from individuals with other neurodegenerative and non‐neurodegenerative diseases.

**Result:**

Here, we present results from analytical validation of the Duritect‐AD^TM^ Test. Previous published results showed that a panel composed of eight autoantibody biomarkers differentiated ADNI participants with confirmed prodromal AD from corresponding age‐ and sex‐matched controls with 96% accuracy, 92% specificity and 100% sensitivity. These same autoantibody biomarkers were also capable of identifying cognitively normal subjects who later converted to MCI or AD, years before the onset of symptoms.

**Conclusion:**

Results demonstrate the utility of the Duritect‐AD^TM^ blood‐based autoantibody test as an accurate, non‐invasive, and inexpensive diagnostic screener, not only for detection of early, AD‐related pathology associated with prodromal AD (MCI), but also at earlier presymptomatic disease stages, several years before the onset of clinical symptoms.

